# Two-Stage Detection Algorithm for Kiwifruit Leaf Diseases Based on Deep Learning

**DOI:** 10.3390/plants11060768

**Published:** 2022-03-13

**Authors:** Jia Yao, Yubo Wang, Ying Xiang, Jia Yang, Yuhang Zhu, Xin Li, Shuangshuang Li, Jie Zhang, Guoshu Gong

**Affiliations:** 1 College of Information Engineering, Sichuan Agricultural University, Ya’an 625000, China; 201902257@stu.sicau.edu.cn (J.Y.); 201902250@stu.sicau.edu.cn (Y.W.); 202005513@stu.sicau.edu.cn (Y.X.); 201902150@stu.sicau.edu.cn (X.L.); 202005907@stu.sicau.edu.cn (S.L.); 2 Sichuan Key Laboratory of Agricultural Information Engineering, Ya’an 625000, China; 3 School of Computing, National University of Singapore, Singapore 119077, Singapore; e0809384@u.nus.edu; 4 College of Agronomy, Sichuan Agricultural University, Chengdu 611130, China; wangjun1@stu.sicau.edu.cn

**Keywords:** deep learning, computer vision, kiwifruit disease detection, smart agriculture

## Abstract

The prevention and management of crop diseases play an important role in agricultural production, but there are many types of crop diseases and complex causes, and their prevention and identification add difficulties to the process. The traditional methods of identifying diseases mostly rely on human visual and manual inspection, which requires a certain amount of expert knowledge and experience. There are shortcomings such as strong subjectivity and low accuracy. This paper takes the common diseases of kiwifruit as the research object. Based on deep learning and computer vision models, and given the influence of a complex background in actual scenes on the detection of diseases, as well as the shape and size characteristics of diseases, an innovative method of target detection and semantic segmentation was proposed to identify diseases accurately. The main contributions of this research are as follows: We produced the world’s first high-quality dataset on kiwifruit. We used the target detection algorithm YOLOX, we stripped the kiwi leaves from the natural background and removed the influencing factors existing in the complex background. Based on the mainstream semantic segmentation networks UNet and DeepLabv3+, the experimental results showed that the ResNet101 network achieved the most effective results in the identification of kiwi diseases, with an accuracy rate of 96.6%. We used the training method of learning rate decay to further improve the training effect without increasing the training cost. After experimental verification, our two-stage disease detection algorithm had the advantages of high accuracy, strong robustness, and wide detection range, which provided a more efficient solution for solving the problem of precise monitoring of crop growth environment parameters.

## 1. Introduction

China is the birthplace of kiwifruit, whose output ranks first in the world [1]. The planting of genetically homogeneous varieties in a large area, resulting in some perennial epidemics such as kiwifruit bacterial canker (*Pseudomonas syingae* pv. *actinidiae*) and kiwifruit brown spot (*Corynespora cassiicola*), has posed a serious threat to the kiwifruit industry. For a long time, the diagnosis of kiwifruit diseases has mostly relied on the visual estimation of plant disease experts for identification and judgment, which is labor intensive and difficult to keep pace with, in terms of real-time monitoring. At the same time, agricultural producers cannot make quantitative and precise analyses and judgments on the degree of damage of the diseases based on the actual conditions of the crops. The research on the detection of kiwifruit leaf diseases is of great significance in agricultural development. It can guide growers to eliminate the pathogens in the budding stage to minimize the loss of kiwifruit. It can also effectively reduce the use of pesticides, making kiwifruit and other agricultural products safer. With the rise of precision agriculture, the use of computer vision technology to detect kiwifruit leaf diseases has occupied an important position.

Compared with traditional inspection methods, computer vision is characterized by fast speed and multiple functions. Computer vision technology is widely used in agriculture [2]. Sun et al. [3] used the YOLOv4 model to detect citrus representation defects on trees by deep learning. Deng et al. [4] used a semantic segmentation method and drones to accurately segment a paddy field. At the same time, with the increase in population and the reduction of arable land caused by the processes of urbanization, the development of agriculture in the direction of high quality and yield has become key. The application of computer vision technology in the field of disease diagnosis can reduce the impact on agriculture to a certain extent and promote the continuous development of agriculture in the direction of high quality and high yield. Wang et al. [5] proposed the best model for rice disease identification based on migration learning. Li et al. [6] used an improved YOLOv3 model to detect diseases on multiple leaves of rose.

It is difficult to process multiple leaves, natural scenes, and diversified diseases for the current model of leaf disease detection. Many studies only focus on a single condition. At the same time, many studies have been conducted on the color characteristics of the lesions, and their texture features have not been used. Wang [7] used the machine vision method to detect rapeseed *Sclerotinia* disease. The research only focused on one single disease. Meng et al. [8] developed a lightweight CNN crop disease recognition model to realize the recognition of multiple diseases, but the development of this research is based on a laboratory environment.

Kiwi canker is a devastating bacterial disease that seriously threatens the production of kiwifruit, known as the “cancer” of kiwifruit [9,10]. Its main characteristics are the appearance of small chlorotic spots and water stains on the new leaves, which later develop into irregular or polygonal brown spots, and there is a wider yellow halo around the diseased spots [9]. Kiwi brown spot is a fungal disease caused by *C. cassiicola* [10]. It is one of the most serious leaf diseases during the growth period of kiwifruit. It has a great impact on yield and fresh fruit quality, and the damage is serious. Its typical symptoms consist of brown circular to irregular target spots with obvious concentric rings appearing on the leaves. The lesions are extended and coalesced, causing leaf necrosis and finally defoliation [9,11]. Brown spot disease and canker disease on leaves are serious and difficult to distinguish in the daily planting process. Therefore, fruit farmers need to detect and intervene as soon as possible to avoid greater economic lose.

To solve the above problems, we collected a high-quality dataset of kiwi fruit leaf diseases, including common plant diseases such as Brown Spot and Bacterial Canker. Based on these diseases, we proposed advanced target detection combined with semantic segmentation to realize a multi-leaf, multi-disease detection model in complex scenes [12]. We used YOLOX [13], the most advanced target detection algorithm in the world, to strip multiple leaves from the natural scene, and place the stripped leaves into our optimized DeepLabV3+ [14] model to accurately detect diseases, and to ensure that both texture and color were used as features to determine the diseases. Finally, ResNet101 [15] was used to achieve accurate recognition.

## 2. Materials and Methods

### 2.1. Experiment Field and Data Acquisition

**Figure 1:**
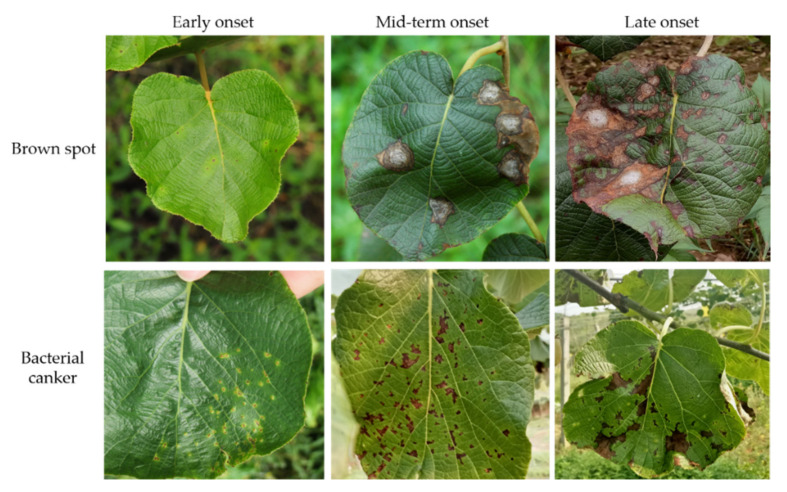
The original picture in the dataset.

The pictures in this study are from the Kiwi Fruit Base in Yaan, Dujiangyan, Sichuan. The dataset was taken by a Canon EOS60D SLR camera with a resolution of 1920 × 1080 pixels, shot by imitating the posture of inspection robot as much as possible. To increase the robustness of the model, we also took pictures from other angles and motion poses. These datasets were taken from March 2018 to August 2021, including disease pictures of different leaf ages, varieties, and onset periods. This dataset marks each kiwi fruit leaf with a clearly visible disease, as Figure 1 shows. After stripping the leaves from the complex nature through target detection, we marked the diseases of the leaves, mainly marking the most similar and common brown spots and cankers. This article collected a total of 1000 images for target detection. After stripping and screening, 2000 datasets with disease were obtained and divided into a training set and validation set at a ratio of 7:3.

### 2.2. YOLOX Background Stripping Algorithm

**Figure 2:**
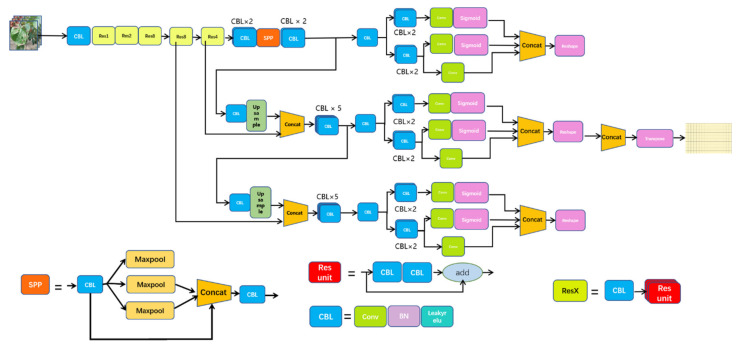
YOLOX network structure diagram.

On the basis of the YOLO series, YOLOX has integrated the latest achievements in the field of target detection in recent years. At the same time, it has inherited the YOLO series, which is easy to deploy, and has made some empirical improvements to construct a new high-performance detector. When choosing the benchmark model of YOLOX, the authors believes that the Yolov4 [16] and Yolov5 [17] series may have some over-optimization from the perspective of the algorithm based on the anchor frame; hence, they finally chose Yolov3 [18] and combined it with the SPP [19] components to develop the Yolov3_spp version with better performance. Based on this, the authors proposed the network structure of YOLOX-Darknet53, as shown in Figure 2.

### 2.3. A Semantic Segmentation Algorithm Based on Axial–DeepLabv3+ Leaf Spots

#### 2.3.1. DeepLab Series Model

Semantic segmentation refers to the classification of each pixel of an image through computer deep learning, which realizes the separation and labeling of different types of objects in the picture. The input picture, GroundTruth, and network output often have the same size. The most representative semantic segmentation models are UNet, SegNet, FCN, PSPNet, DeepLab, etc. [20,21,22,23].

DeepLab is a semantic segmentation model that has good performance on public datasets such as VOC. Among them, the DeepLabV3+ model is the current DeepLab model with better effectiveness. We used a variety of semantic segmentation models to conduct experimental comparisons on the kiwi leaf dataset, including DeepLabV1, DeepLabV2, DeepLabV3 and DeepLabV3+, and finally achieved a significant improvement in accuracy.

**Figure 3:**
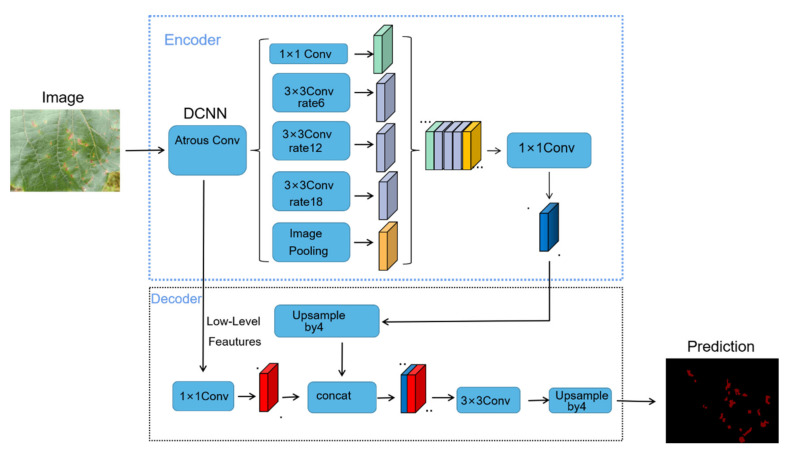
DeepLabv3+ network structure diagram, where, .., … are the symbol of omission.

DeepLabV1 is an improvement of the VGG network. It tries to fuse multi-level information by connecting to the convolutional layer after the Maxpool layer. DeepLabV2 mainly introduces atrous spatial pyramid pooling (ASPP) on the basis of DeepLabV1 to enhance the model’s ability to recognize objects of the same category of different sizes. On the basis of DeepLabV2, DeepLabV3 adds a hole convolution of different rates in the back end of the model and introduces batch normalization in ASPP. DeepabV3+ adjusts the structure of DeepLabV3 to form an encoder and decoder similar to U-net, allowing the model to achieve better results at the edge of segmentation. The modified Xception is then introduced to enhance the robustness of the model classifier [24]. The DeepLabV3+ model structure is shown in Figure 3.

#### 2.3.2. Attention Mechanism in DeepLabv3+ Series Models

Axial–DeepLabV3+ introduces the Axial–Attention module in DeepLabV3+ to achieve a better attention mechanism effect while ensuring that the parameters are within an acceptable range [25].

**Figure 4:**
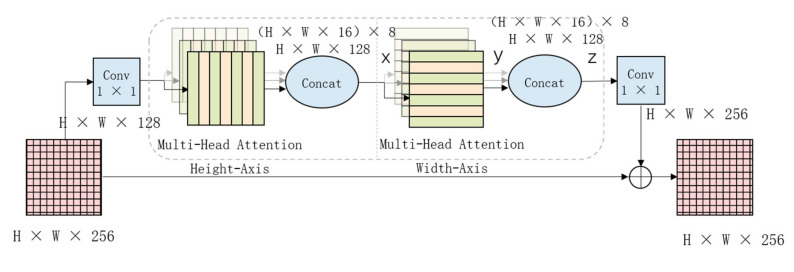
The module of axial.

Therefore, in the experiment, we introduce this module into DeepLabv3+ to increase the model’s attention to the injury area and to ensure the accuracy of the model’s identification.The schematic diagram of the module is shown in Figure 4.

#### 2.3.3. Selection of Backbone of DeepLabV3+ Model

We tried the three classifiers, Xception and MobileNet [26], and ResNet101 proposed by DeepLabV3+ and compared the models in consideration of the amount and accuracy of the model parameters in order to determine the optimal classifier to improve the injury recognition accuracy of classification.

The Backbone selection process also plays the role of accurate rate comparison with the direct image classification algorithm to reflect the performance of the semantic segmentation model for the classification of plant leaf injuries, and at the same time exploring the best plant leaf injury recognition and classification method.

#### 2.3.4. Attempt and Optimization of Dice Loss and Focal Loss Function

Choosing a suitable loss function is conducive to the improvement of the accuracy of the model. The experiment compares two loss functions: Focal Loss and Dice Loss.

(1) Focal loss: In the classification process, the background class is often easy to classify but difficult to classify different types of injuries. Therefore, the classification difficulty varies [27], which is suitable for optimization through Focal Loss. When the number of negative samples is large, it accounts for most of the total loss, and most of them are easy to classify, such that the optimization direction of the model is not as expected, and we can control the shared weight and control of loss by positive and negative samples. The weights of easy-to-classify and difficult-to-classify samples are used to optimize the loss function. After optimization, the loss function is as follows, where $p_{t}$ represents the predicted value of the model, and γ and $\partial_{t}$ are two factors based on the standard cross entropy loss function:

(1) $$FL\left(pt\right)=-\partial t{\left(1-pt\right)}^{\gamma }\log\left(pt\right)$$

(2) Dice Loss: It is observed that there is a large gap between the background and the ratio of injuries, which applies the loss function optimization through Dice Loss [28]. Dice Loss can be defined as follows, where $\left|A\cap B\right|$ represent the number of common elements of *A* and *B*. $\left|A\right|$ and $\left|B\right|$ represent the number of elements in each collection:

(2) $$Dice=\frac{2|A\cap B|}{\left|A|+|B\right|}$$

It can increase the impact of leaf injury area on the loss function, thereby increasing the accuracy, robustness and applicability of the model.

#### 2.3.5. Learning Rate Decay

In order to prevent the learning rate from being too large, in which condition it will oscillate back and forth when it converges to the global best point, the learning rate should be continuously reduced as the epochs grow. In addition, the learning step size of the convergence gradient should be reduced in order to achieve more stable and accurate training results.

Commonly used learning rate decay strategies include exponential_decay, natural exp decay, cosine decay, etc. We used noisy linear cosine decay, which is often used in reinforcement learning and in semantic segmentation model training to study its role in the field of computer vision [29].

Noisy_linear_cosine_decay adds noise to the decay process on the basis of linear cosine decay, which increases the randomness and possibility of finding the optimal value of lr to some extent. It is also an improvement on cosine decay, and its calculation formula is as follows, where $\xi_{t}$ stands for random noise factor, and ld and cd stand for factors controlling the gradual decline of learning rate. Equation (2) represents noisy linear decay. $l_{new}$, $l_{init}$, and $l_{min}$ stand for learning rate of this epoch, learning rate of the beginning, and minimum learning rate, respectively. $T_{max}$ stands for the maximum epoch.

(3) $$\xi t\sim N\left(0,1/{\left(1+t\right)}^{0.55}\right)$$

(4) $$\left(ld+\xi t\right)\;\times \;cd+0.001$$

(5) $$l_{new}=l_{min}+(l_{init}-l_{min})\times (1+\cos(\frac{epoch}{T_{max}}\pi ))$$

#### 2.3.6. Implementation and Evaluation Index

**Figure 5:**
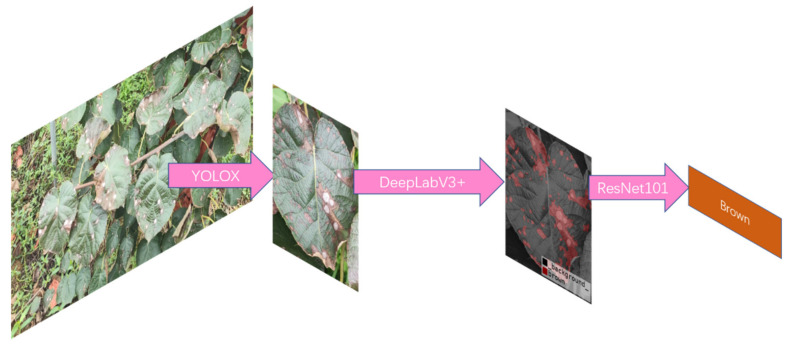
Overall processing flow of the network.

Based on the above analysis, we innovatively proposed a two-stage leaf disease recognition algorithm. The algorithm flow-chart is as Figure 5 follows.

The training of the model was completed using Windows 10 operating system and Pytorch framework. The CPU model of the test equipment was Intel^®^Core™ i9_10900K CPU@3.70 GHz, the GPU model was GeForce RTX 5000 16 G, and the software environment was CUDA 10.1, CUDNN 7.6, Python3.7. All experiments were trained with default parameters.

This paper introduced Precision (*P*), namely precision rate, recall rate (Recall, *R*), and Mean Average Precision (*mAP*) to evaluate the performance of the kiwi defect detection model. The expressions of *P* and *R* are as follows:

(6) $$P=\frac{TP}{\left(TP+FP\right)}$$

(7) $$R=\frac{TP}{\left(TP+FN\right)}$$

Among them, *TP* (true positive), *FP* (false positives), and *FN* (false negatives) respectively represent positive samples with correct classification, negative samples with incorrect classification, and positive samples with incorrect classification.

*AP* is the average accuracy rate, which is the integral of the *P* index to the *R* index, that is, the area under the *PR* curve; *mAP* is the mean average accuracy, which means taking the average value of *AP* of each category. They are defined as follows:

(8) $$AP=\int_{0}^{1}P\left(R\right)dR$$

(9) $$mAP=\frac{1}{\left|Q_{R}\right|}\underset{q=Q_{R}}{\sum }AP\left(q\right)$$

## 3. Results

### 3.1. Experimental Results

YOLOX is used to separate the pictures from the natural scenes such that subsequent models can accurately identify diseases.

*mAP* is used to measure the quality of the defect detection model. The higher the value, the higher the average detection accuracy of the model and the better the performance of the model for blade recognition. The *mAP* of training and the loss of a valid dataset are in the figures below.

**Figure 6:**
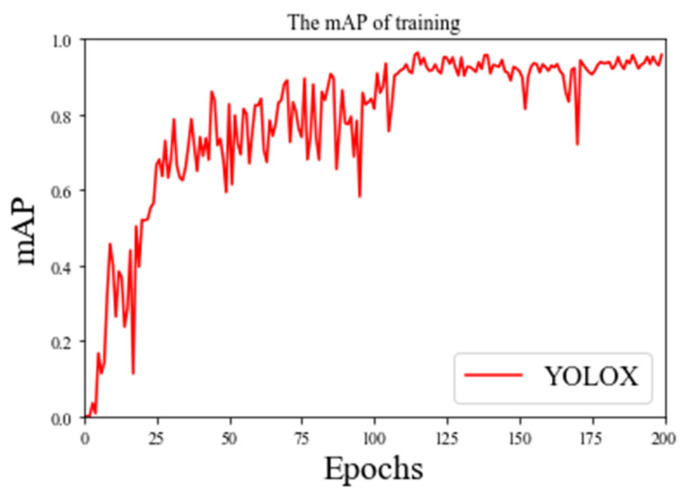
The *mAP* of training.

**Figure 7:**
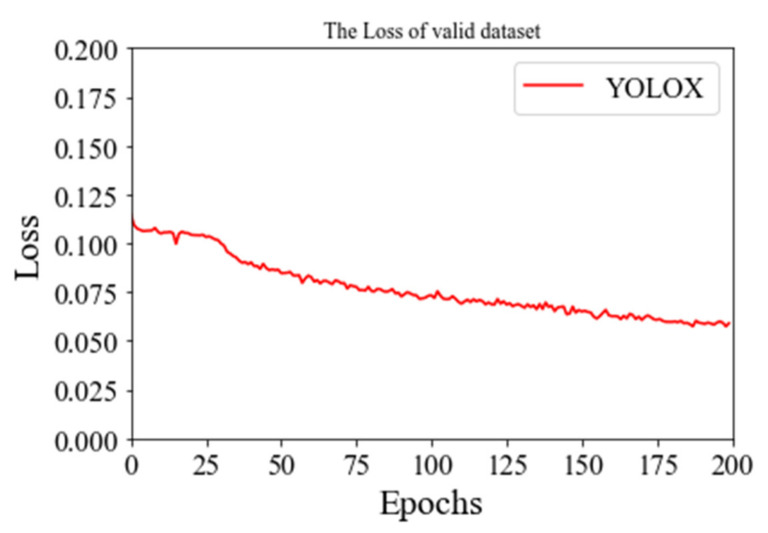
The loss of a valid dataset.

**Figure 8:**
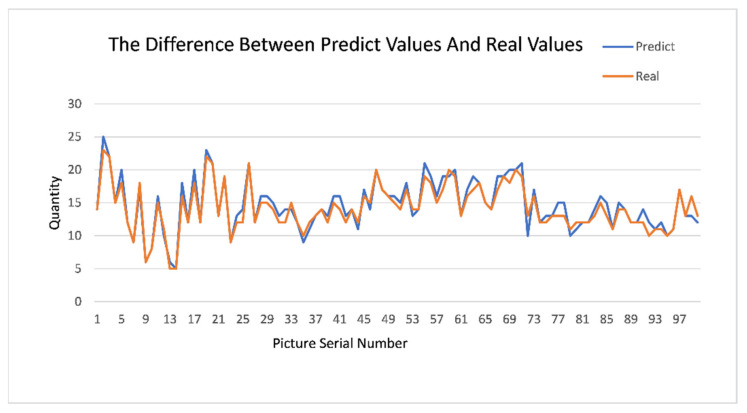
The difference between predict values and real values.

As can be seen from the Figure 6 and Figure 7, YOLOX’s *mAP* of leaf detection reaches 95%, from which we believe that YOLOX has been accurately removed from the complex natural environment. In order to prove the effectiveness and robustness of the model, we introduced 100 pictures of natural images of leaves that did not participate in the training. Then, we manually counted the effective leaves in the pictures, detected them by the model and plotted the difference between the predicted values and the real values, which showed that the missed detection rate was 0.02. The comparison is shown Figure 8.

**Figure 9:**
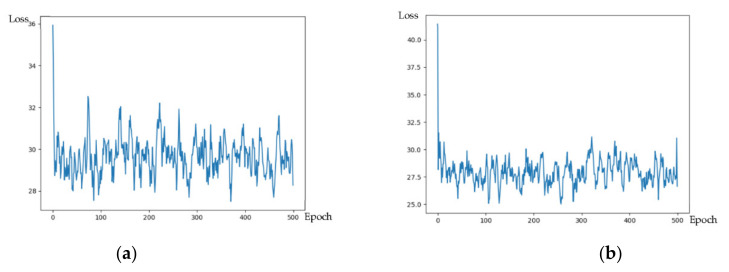
Comparison of loss before and after improvement. (**a**) Loss of DeepLabv3+. (**b**) Loss of our DeepLabv3+.

After obtaining the stripped leaves, we carried out the precise segmentation of diseases and conducted experiments and improvements on the UNet and DeepLab series of networks. The actual training process of our improved DeepLab model and the original model is compared as Figure 9 follows: the left is the original model, and the right is the improved model. The volatility and loss values were reduced, indicating that the improvement is more effective.

It can be seen from the training loss graph analysis that the improved model converges faster with less oscillation on each epoch and with better effectiveness.

**Figure 10:**
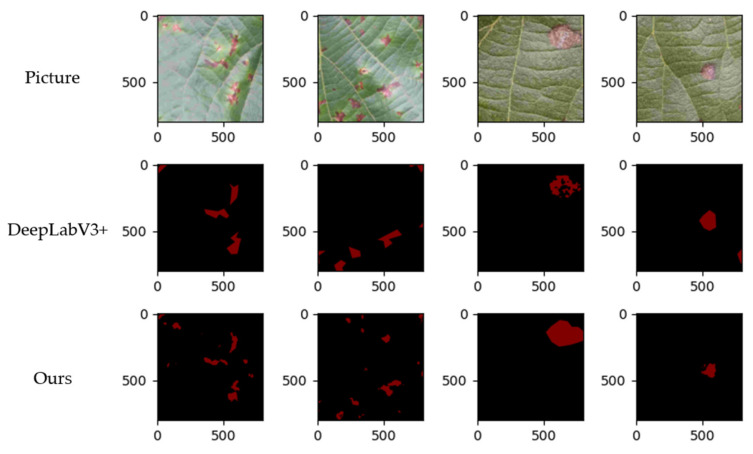
Comparison of performance.

Figure 10 shows after the segmentation, where the first behavior in the picture is based on the original model and the second behavior is based on the improved model. The segmentation accuracy of the picture is higher, indicating that the newly selected loss function can handle the loss more effectively and achieve a better segmentation effect.

### 3.2. Analysis

During the segmentation process, we compared the combination of multiple network structures and training methods and obtained the following Table 1 experimental results.

After the innovation experiment of the U-Net series models, we followed up with the DeepLab model test to ensure the credibility of the experimental results. In the process of the experiment, we mainly compared the improvement of the model training accuracy by different training methods. As Table 2 shows:

We selected the last group of training strategies, and based on this, we improved the deep labv3+ model and obtained the following experimental comparison results. As Table 3 shows:

After the experiments and model comparisons, we finally determined that the training methods of DeepLabV3+ and ResNet + Focal Loss + cosine_decay were the models with the highest accuracy. In addition, the combination of UNet++ and Focal Loss can reach good accuracy performance while reducing the number of parameters.

We determined that the final segmentation model was DeepLabV3 along with Focal Loss, attention gates, and noisy linear cosine decay + ResNet101, with final recognition accuracy reaching 96.6%. Compared with the original deeplabv3 + network with an accuracy of 95.6%, the accuracy improved by 1.0%.

## 4. Discussions

This article explored a method to detect kiwifruit leaf diseases. To realize the need to detect kiwifruit diseases from complex natural scenes, we used the YOLOX model for more in-depth research. To accurately detect diseases, we used DeepLabv3+ to cut the disease parts off the leaves. Finally, a high-precision classifier was used to identify the diseases. The feasibility of this method is as follows.

(1) In terms of data, we tried to imitate the perspective and posture of the detection robot as much as possible; hence, our algorithm had strong practicability. After the investigation, we found that the brown spot and bacterial canker of the kiwifruit had high incidence and infectiousness. Therefore, to solve the urgent need of fruit farmers, we used only the brown spot and canker training models to speed up the research progress.

(2) In terms of the model generalization ability, we collected pictures of different species, onset periods, and leaf ages. At the same time, YOLOX adopted mosaic and mixup data enhancement strategies, effectively improving the model’s generalization ability and robustness strategy.

(3) In DeepLabv3+ model optimization, we introduced the axial–attention module to gain better experimental results, but the number of parameters increased, which was derived from the higher resolution pictures in the case of a relatively low injury rate. Its position tended to be close to the center of the page. For pictures with a relatively large amounts of injuries, attention gates made it easier to identify injuries and made the model perform better on the test set data. Thus, we think this was an effective innovation. In our experiment, it was difficult to distinguish certain classes, and Focal Loss had better performance and more accurate segmentation. In this experiment, Focal Loss performed better.

(4) In the model training method, we introduced the noisy linear cosine decay method for training and improved the training effect as much as possible without increasing the training cost.

In summary, we achieved two stages to strip the leaves from the complex scene, and then accurately cut the diseased spots, and finally, we accurately identified the diseased spots through the classifier. Therefore, we believe that this research provides better quality for the natural scene identification of diseases. The strategy is an exploration of great significance.

## 5. Conclusions and Future Work

To realize the detection of leaf diseases of kiwifruit based on natural scenes, this paper proposed a two-stage detection method. Using YOLOX to strip the leaves from the complex natural scenes, we could detect the leaves with an accuracy of up to 95%. Then, we optimized the mainstream semantic segmentation network DeepLabv3+ to accurately cut the lesions. Furthermore, we introduced the axial–attention module to shield some interference information and leveraged Focal Loss as our loss function to accurately segment the lesions. Finally, the ResNet classifier was used to identify lesions, and the accuracy plus 1.0% was compared with the original DeepLabv3+ model, which had better robustness and which proved the effectiveness of the model.

This paper mainly proposed a segmentation high-precision detection algorithm for leaf diseases. However, there are still problems regarding fewer diseases and slower identification. In the future, we will add more diseases and lack-of-element data for training. In addition, the algorithm proposed in this paper had good performance, but the detection speed had difficulty meeting the requirements of real-time identification of kiwifruit leaf disease. Follow-up research can reduce the complexity of the model by pruning the network, achieving parallel speed and accuracy, and using it as soon as possible in the field of agricultural disease control.

## Figures and Tables

**Table 1:** U-net series model comparison.

Method	Loss Function			Learning Rate Decay	Test Accuracy
	Dice Loss	Focal Loss	Cross Entropy Loss		
UNet			√		0.951
Attention UNet			√		0.950
UNet++			√		0.953
UNet++		√			0.952
UNet++	√				0.953
UNet++	√			Cosine Decay	0.954
UNet++	√			Noisy linear cosine decay	0.956

Where √ means we used this strategy, blank means we don’t used.

**Table 2:** Comparison of training skills of DeepLab series models.

Method	Loss Function			Learning Rate Decay	Test Accuracy
	Dice Loss	Focal Loss	Cross Entropy Loss		
DeepLabV1			√		0.943
DeepLabV2			√		0.951
DeepLabV3			√		0.955
DeepLabV3+			√		0.956
DeepLabV3+		√			0.957
DeepLabV3+	√				0.956
DeepLabV3+		√		Noisy linear cosine decay	0.959

Where √ means we used this strategy, blank means we don’t used.

**Table 3:** Comparison of DeepLabv3 + model optimization.

Base Model	Backbone			Attention Gates	Test Accuracy
	Xception	MobileNet	ResNet101		
DeepLabV3+		√			0.954
DeepLabV3+	√				0.959
DeepLabV3+			√		0.961
DeepLabV3+			√	√	0.966

Where √ means we used this strategy, blank means we don’t used.

## Data Availability

The data presented in this study are available upon request from the corresponding author. The data are not publicly available due to our new research is also using these data.
